# Supplementary material for: One fly–one genome: chromosome-scale genome assembly of a single outbred Drosophila melanogaster
Source: Nucleic Acids Res. 2020 Jun 3;48(13):e75. doi: 10.1093/nar/gkaa450 (PMC7367183; doi:10.1093/nar/gkaa450)
Supplement: gkaa450_Supplemental_File [file gkaa450_supplemental_file.pdf]

Supplementary Materials

to

One fly - one genome : Chromosome scale genome assembly of a single *drosophila melanogaster*

by

Matthew Adams, Jakob McBroome, Nicholas Maurer, Evan Pepper, Nedda F. Saremi, Richard E. Green, Christopher Vollmers, Russel Corbett-Detig

Table of contents

Supplementary Figure S1. Coverage depth

Supplementary Figure S2. Wolbachia contact map

Supplementary Table S1. Repeat content

Supplementary Table S2. Polishing

Supplementary Table S3. Chromosome Y coverage

A

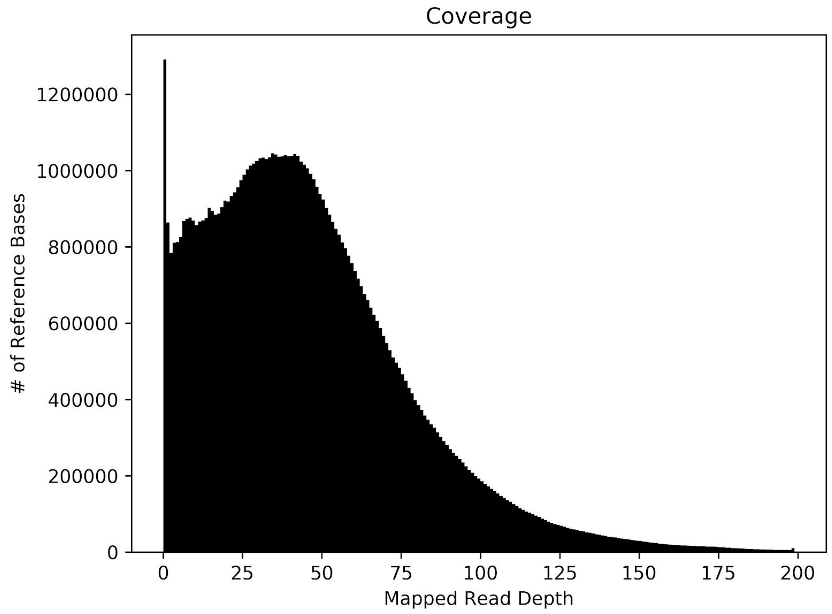

B

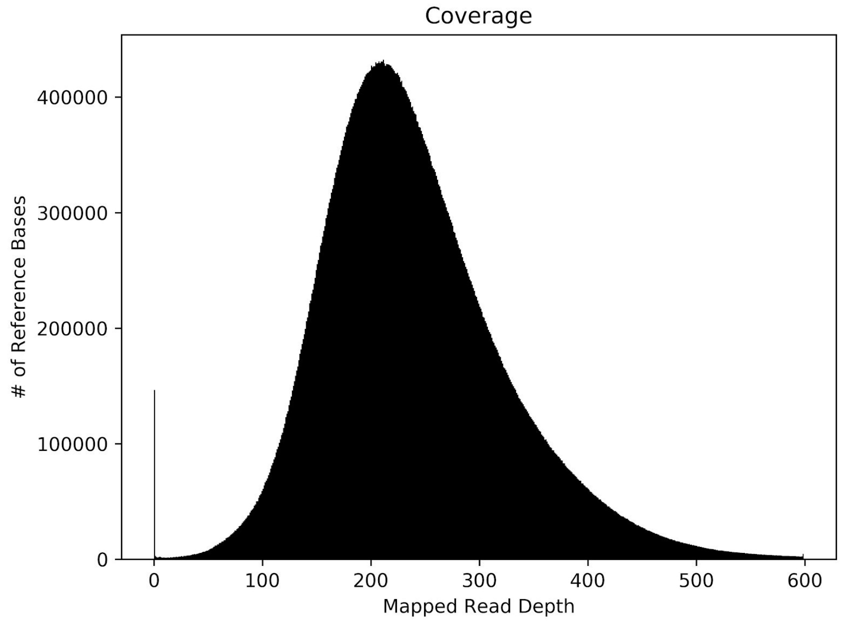

Supplementary Figure S1: Coverage depth. Sequencing coverage depth of primary assembly data mapped to the dm6 reference genome (A) long-read data set (B) short-read data set. Generated by aligning raw reads to the reference genome using Minimap2 then samtools depth function to calculate depth of sequencing per base of autosomes.

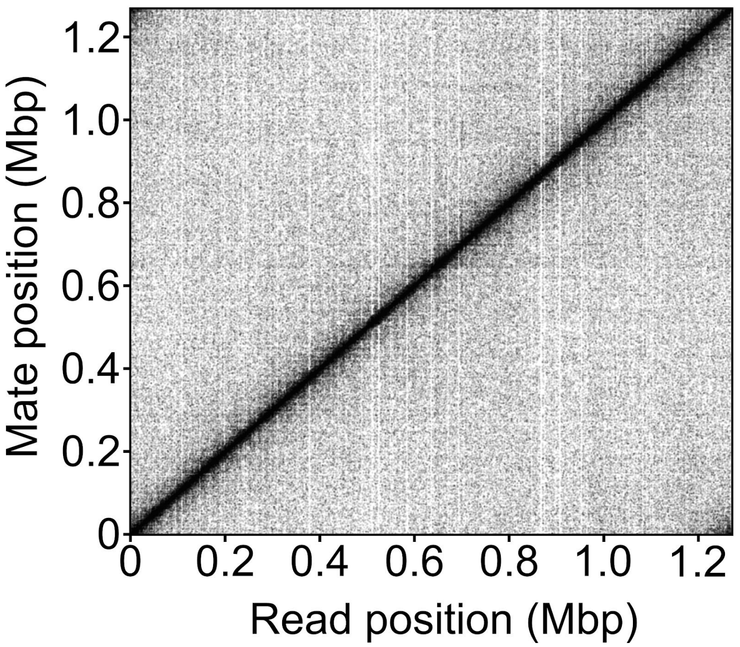

Supplementary Figure S2. Wolbachia Hi-C contact map.

	Pilon0		Pilon1		Pilon2		Pilon3		Pilon4		A4	
Element Type	Mean coverage	Percentage with minimum 90% coverage	Mean coverage	Percentage with minimum 90% coverage	Mean coverage	Percentage with minimum 90% coverage	Mean coverage	Percentage with minimum 90% coverage	Mean coverage	Percentage with minimum 90% coverage	Mean coverage	Percentage with minimum 90% coverage
Mobile genetic element	0.57	52.42	0.59	54.00	0.60	55.02	0.60	55.02	0.61	55.29	0.89	82.03
Gene	0.91	87.92	0.92	88.42	0.92	88.73	0.92	88.73	0.92	88.83	1.10	97.95
lncRNA	0.92	90.06	0.93	90.10	0.93	90.72	0.93	90.72	0.93	90.68	0.99	98.77
Exon	0.94	93.18	0.94	93.54	0.94	93.81	0.94	93.81	0.94	93.88	1.01	99.54
mRNA	0.93	86.70	0.93	87.43	0.93	87.81	0.93	87.81	0.93	88.05	1.02	97.40
CDS	0.94	93.34	0.94	93.69	0.94	93.93	0.94	93.93	0.94	94.00	1.00	98.97
Primary transcript	0.92	91.63	0.92	92.01	0.93	92.40	0.93	92.40	0.93	92.39	1.66	99.23
miRNA	0.89	92.48	0.89	92.69	0.90	93.52	0.90	93.52	0.90	93.74	1.30	99.58
Pseudogene	0.42	36.69	0.44	37.99	0.44	38.31	0.44	38.31	0.44	38.31	1.08	71.42
tRNA	0.77	74.61	0.80	76.91	0.79	76.48	0.79	76.48	0.79	76.49	0.98	98.75

Supplementary Table S1: Coverage By Element Type. Full table of coverage values for four assemblies for all annotated elements with a total count in the melanogaster genome above 50.

	Assembly size (mb)	N50 (mb)	# misassemblies	# mismatches per 100 kbp	# indels per 100 kbp
**Pre-polishing**	111.36	26.182	777	671.83	163.30
**1st round**	111.84	26.24	802	556.68	96.31
**2nd round**	112.02	26.26	802	532.23	92.40
**3rd round**	112.14	26.272	800	524.78	89.12
**4th round**	112.22	26.279	798	525.36	88.77

Supplementary Table S2: Polishing. Brief summary of QUAST genome assembly metrics from four iterative rounds of polishing using Pilon.

	ChrY sequencing coverage	ChrY:Autosome average sequencing depth
**ONT R2C2 library**	20 %	2.3x : 46x
**Illumina Tn5 library**	44 %	40x : 244x
**Illumina Hi-C library**	37 %	19x : 132x

Supplementary Table S3: Chromosome Y coverage. Coverage of the Y chromosome. Generated by aligning raw reads to the dm6 reference genome using Minimap2 then samtools depth function to determine the percent of Y chromosome coverage and depth of sequencing per base of the Y chromosome.
